# Disseminated Varicella-Zoster Virus Presenting With Pneumonitis and Diffuse Alveolar Hemorrhage in an Immunocompetent Patient

**DOI:** 10.7759/cureus.66895

**Published:** 2024-08-14

**Authors:** Abhinav K Rao, Thomas J Lee, Hassan Khokhar, Sokol Kalaveshi

**Affiliations:** 1 Internal Medicine, Trident Medical Center, North Charleston, USA; 2 Internal Medicine, Rutgers New Jersey Medical School, Newark, USA; 3 Internal Medicine, HCA Florida Orange Park Hospital, Orange Park, USA

**Keywords:** acute respiratory distress syndrome (ards), visceral varicella zoster, varicella-zoster, disseminated varicella, vzv

## Abstract

Varicella pneumonitis is typically seen in individuals with risk factors such as male gender, smoking history, and immunocompromised state and is often associated with disseminated infection, whereas primary varicella-zoster virus (VZV) infection usually involves a diffuse vesicular rash and rarely progresses to viral pneumonia. VZV pneumonitis accompanied by disseminated VZV infection is associated with a high mortality rate and may progress to diffuse alveolar hemorrhage in severe cases. In addition to cutaneous lesions, patients typically develop dyspnea, cough, tachypnea, chest pain, fever, and hemoptysis. Here, we present a rare case of disseminated VZV infection in an immunocompetent patient with pneumonitis and diffuse alveolar hemorrhage.

## Introduction

Varicella pneumonitis in adults has an incidence range of 5-15%. It usually occurs in individuals with underlying lung disease, immunosuppression, pregnancy, or smokers [1,2]. Within the cohort of immunocompetent patients, its incidence is thought to be even less, estimated to be seen in 1/400 patients [2]. The pneumonitis typically develops one to six days after the development of skin lesions and can be rapidly progressive, resulting in diffuse alveolar hemorrhage in severe cases [3,4]. Importantly, mortality rates of varicella-zoster virus (VZV) pneumonitis can reach up to 24% in critically ill patients [4].

## Case presentation

We present the case of a male in his 30s, with no significant past medical history, who presented with two days of progressive symptoms of pleuritic pain, fever, and chills combined with erythematous rash, which first started as a pinpoint on his chest and evolved into covering his torso and proximal upper and lower extremities. He had no prior history of VZV infection. He denied any significant family history. He denied any unprotected sexual contact or any sick contact. He works as a machine operator. The patient is a native of Nicaragua who emigrated five years ago to the United States. He has not had any recent travel in or out of the country during the last six months. He continues to smoke approximately two cigarettes a day (first started approximately 15 years ago). He denies alcohol use, illicit intravenous drug use (IVDU), or any other recreational drugs. His only new medication was Tylenol, which he took for the fever. He does not take any other medications or supplements.

**Figure 1:**
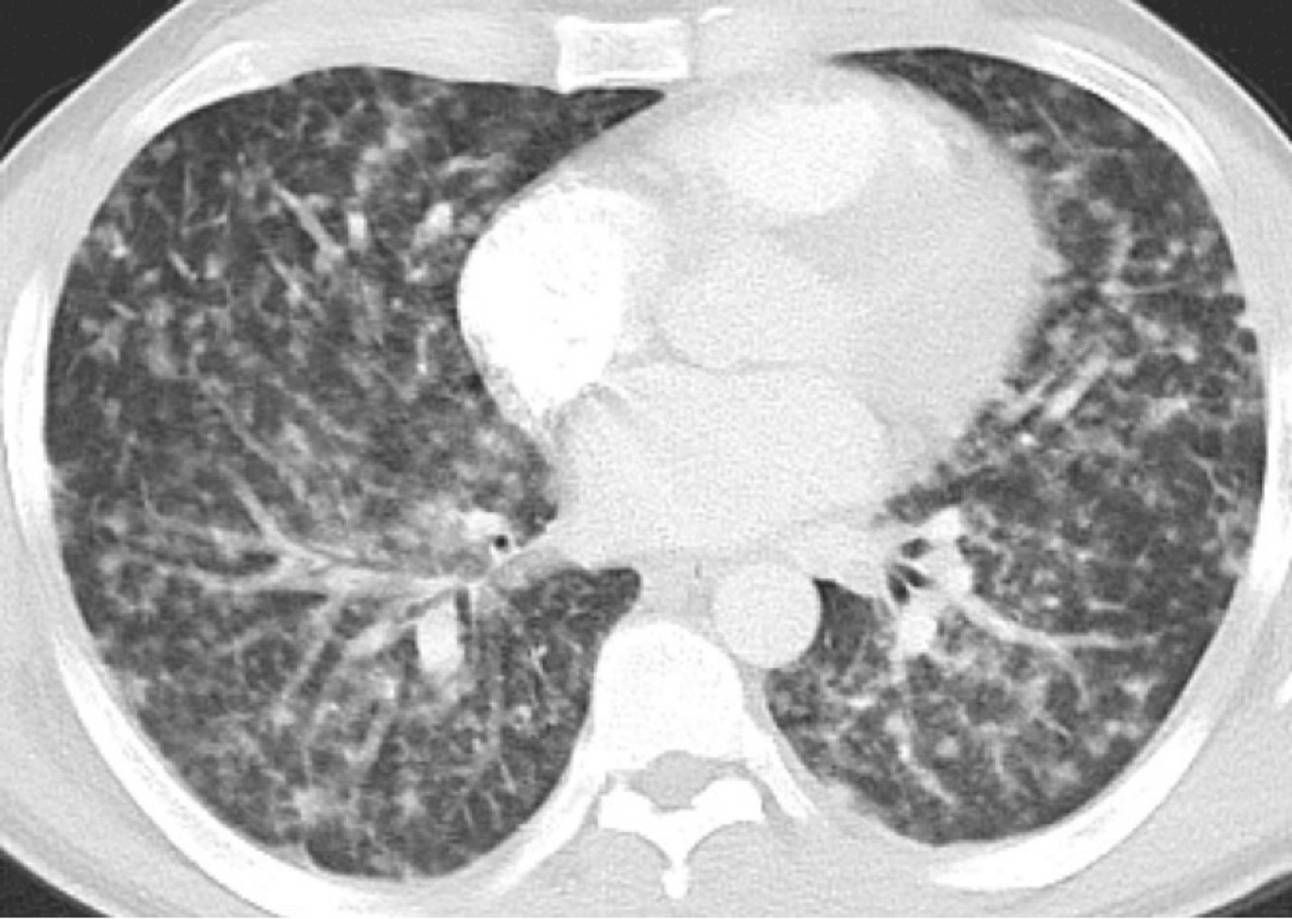
Disseminated VZV with diffuse bilateral nodular consolidation Extensive bilateral areas of nodular consolidation on the presentation ultimately developed into diffuse ground glass disease

His vitals upon arrival were as follows: heart rate of 100 beats/min, respiratory rate of 18 breaths/min, blood pressure of 113/75 mmHg, and SaO_2_ of 97% on room air. On physical exam, he was lethargic and intermittently responding to questions. From a respiratory standpoint, the patient's breathing was labored. Cardiac auscultations showed a regular rate and rhythm with no murmurs or gallops. He was in respiratory distress with a rapid increase in oxygen requirement from nasal cannula to heated high flow to intubation within a span of six hours. Diminished breath sounds and scattered crackles were noted throughout all lung fields. No wheezing was noted. His skin exam revealed a diffuse pustular rash with vesicles on his face, trunk, and proximal extremities. The patient's laboratory data are summarized in Table 1. Chest X-ray (CXR) was performed, which showed multifocal pneumonia. Subsequent computed tomography of his thorax revealed extensive bilateral nodular consolidation (Figure 1).

**Table 1:** Laboratory data on admission

Parameters (Reference Range)	Patient Values
Sodium (136–145 mEq/L)	135
Potassium (3.6–5.1 mEq/L	3.5
Chloride (101–111 mEq/L)	102
Carbon Dioxide (22–32 mEq/L)	25
Anion Gap (3–13)	8
BUN (6–20 mg/dL)	11
Creatinine (0.7–1.2 mg/dL)	1.0
Glucose (70–100 mg/dL)	112
Lactic Acid (0.5–2.0 mEq/L)	2.1
Calcium (8.9–10.3 mg/dL	8.1
Total Bilirubin (<1.0 mg/dL)	0.9
AST (<35 units/L)	135
ALT (10–63 units/L)	156
Alkaline Phosphatase (32–101 units/L)	79
Troponin (<0.04 ng/mL)	<0.03
Total Protein (6.–8.0 gm/dL)	6.4
Albumin (3.4–4.8 gm/dL)	4.1
Coagulation	
D-Dimer (0–499)	5133
Hematology	
WBC (4.0–10.9 k/mm^5^)	5.3
Hgb (13.5–16.5 g/dL)	16.0
Plt Count (135–350 k/mm^3^)	78

In the emergency room, he received 1 L intravenous fluids (IVFs), azithromycin, and 60 mg of IV solumedrol presuming CTA findings were related to alveolar hemorrhage. He was initiated on ceftriaxone. Over the span of three hours since arriving at the emergency room, the patient's oxygen requirement rapidly increased to 15 L to a heated high-flow nasal cannula (HHFNC) of 60 L/100% FiO_2_. Given his rapid increase in oxygen requirement and worsening mental status, the decision was made to intubate him. In the ICU, he developed acute respiratory distress syndrome (ARDS), and his hemodynamic status progressed to septic shock. On the second day of ICU stay, he required norepinephrine and vasopressin, which were continued for seven days (peak requirement: norepinephrine 12, vasopressin 0.04).

The patient experienced rapidly progressive respiratory deterioration, which was atypical of an infection. However, given his fevers, rash, and consolidation noted on the CT chest, VZV infection was certainly a consideration. Viral infection, particularly disseminated VZV, was high on the differential given his cutaneous lesions. Additionally, a full serological autoimmune and connective tissue disease work was ordered. However, he had no prior history or family history of autoimmune disease. Sarcoidosis was another important consideration given his bilateral airspace disease, granulomatosis disease on CT, hemoptysis, and rash. Finally, early ARDS was also considered, as his findings on CT imaging and rapidly increasing oxygen requirement were consistent.

On his third day of hospitalization, he was started on IV gancyclovir 475 mg Q12H due to high suspicion of disseminated VZV infection. The patient continued to have high oxygen requirement and was treated with a four-day course of dexamethasone (10 mg IV on day one, followed by 5 mg IV for three days), with clinical improvement evidenced by a decrease in oxygen and pressor requirement, as well as containment and crusting of his rash. Bronchoscopy was also performed, which showed diffuse, thick, bloody secretions extending from the mainstem bronchi distally into all visualized airways and resulting in plugging of smaller subsegmental airways. These were cleared with suctioning and lavage. Of note, bronchial membranes were friable and remained persistently bloody with modest clearing with serial aliquots, suggesting bland capillaritis. The patient was also given spot diuresis during his ICU course. His antibiotics were transitioned to Ancef for bilateral multifocal pneumonia secondary to methicillin-susceptible Staphylococcus aureus (MSSA) on day five, for which he completed a seven-day course. The patient was eventually extubated on day 14 to heated high flow and eventually transitioned to a 3 L nasal cannula.

**Figure 2:**
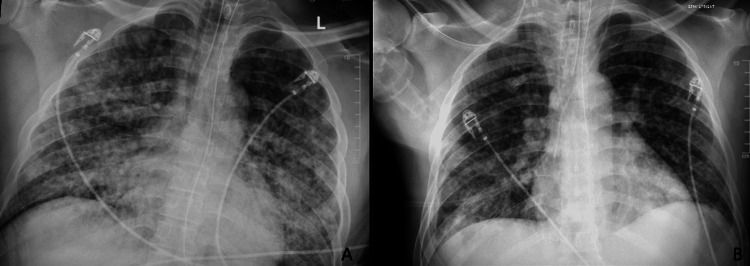
Improvement in bilateral consolidation after treatment of disseminated varicella-zoster virus (VZV) Plain films on admission (A) and 12 days after admission (B), showing greatly improved bilateral consolidation.

He was transferred out of the ICU after 15 days to the general medical floor. Serologies eventually returned positive for VZV IgG and IgM, which, in the setting of improvement since switching to gancyclovir and adding steroids, were highly suggestive of disseminated VZV infection. The following infectious serologies were negative: syphilis Ab, influenza A and B, COVID, group A strep, MRSA PCR, BAL culture, cryptococcal Ag, HSV 1 and 2 swabs, histoplasma Ag, hepatitis panel, viral respiratory PCR panel, HIV AB+ RNA, and Fungitell. Additionally, his ANA screen, AntidsDNA, RF screen, and anti-GBM were negative. His QuantiFERON-TB Gold test returned positive for tuberculosis (TB), indicating latent TB as his clinical picture was not reflective of active TB. This assessment was supported by imaging findings and a seven-year residency in the United States. At discharge, his skin lesions had crusted over. Follow-up imaging with CXR 12 days after hospitalization showed significant improvement in bilateral airspace disease (Figure 2). The patient had an improvement in oxygen saturation and no further requirement for oxygen supplementation. He was eventually discharged after completing a two-week course of IV ganciclovir.

## Discussion

Varicella pneumonitis in adults has an incidence range of 5-15% usually occurring in individuals with underlying lung disease, immunosuppression, pregnancy, or smokers [1-4]. When severe, the pneumonitis can progress to diffuse alveolar hemorrhage, which in this case was secondary to capillaritis. A single-center retrospective analysis was conducted of immunocompetent adult outpatients with varicella for approximately 19 months to assess progression to VZV pneumonia. Of the 193 immunocompetent adults diagnosed with varicella vesicular rash, 186 patients were found to have CT chest evidence of pneumonia, resulting in an incidence of 70.9% with a mortality rate between 11% and 20% [2]. Newer retrospective studies have shown that incidence is much higher than previously believed, with one study showing an incidence of ~71% (cutaneous and CT evidence of pneumonia), while other studies have reported incidence of 5-50% and high mortality rates ranging from 24-29% [2,3,5].

Studies have shown that patients diagnosed with varicella pneumonitis treated with antivirals including acyclovir, valaciclovir, ganciclovir, and famciclovir administered daily for approximately 7-10 days improved prognosis, contributed to regression of pulmonary calcifications/nodules, and reduced mortality [1,3].

On day three of hospitalization, treatment with ganciclovir was started due to suspicion of disseminated VZV infection. Unfortunately, this patient had progressed to ARDS and septic shock due to rapidly progressing VZV pneumonitis and the presence of coinfection with multifocal MSSA pneumonia. The development and pathogenesis of ARDS due to VZV for the majority is unknown. The ensuing lung injury and the proposed mechanism were theorized as involving neutrophil activation induced increased alveolar epithelial/endothelial-barrier (EEB) permeability, leading to an inundation of protein-rich edema into the alveoli (6), ultimately disturbing alveolar architecture, type II cell hyperplasia, leading to diffuse alveolar damage/hemorrhage [6,7].

Previous studies acquired from clinical cases and autopsies have shown that patients with concomitant disseminated dermal and mucosal vesicular lesions have been found to have necrotic and hemorrhagic areas distributed in both upper and lower respiratory tract and likely have the potential to precipitate bacterial co-infections (2). In our case, bronchoscopy had shown friable bronchial mucosa throughout the upper and lower bronchial tree, which was consistent with diffuse alveolar hemorrhage secondary to capillaritis. In addition to antiviral therapy, though the role of systemic steroids has been controversial in the management of VZV pneumonitis, there are several cases of disseminated VZV that show clinical improvement after systemic steroid administration [4,8,9]. Systemic steroid therapy ultimately decreases ARDS-related intrapulmonary inflammation but also leads to immunosuppression, allowing further viral replication and promoting bacterial superinfection. In our case, the addition of systemic steroids (IV dexamethasone) improved airspace disease and reduced the time requiring invasive mechanical ventilation.

## Conclusions

VZV pneumonitis in immunocompetent adults progressing to ARDS requiring invasive mechanical ventilation is rare and life-threatening. Physicians should start early antiviral therapy in patients who present with severe respiratory and cutaneous lesions as the risk of rapid respiratory decline is significant. Based on our case, early IV antivirals and systemic steroids may lessen ICU course and improve morbidity in the treatment of disseminated VZV.
